# The effect of supplemental arginine on the gut microbial homeostasis of broilers during sub-clinical necrotic enteritis challenge

**DOI:** 10.3389/fphys.2024.1463420

**Published:** 2024-09-17

**Authors:** Shahna Fathima, Walid G. Al Hakeem, Revathi Shanmugasundaram, Jeferson Lourenco, Ramesh K. Selvaraj

**Affiliations:** ^1^ Department of Poultry Science, University of Georgia, Athens, GA, United States; ^2^ Toxicology and Mycotoxin Research Unit, U.S. National Poultry Research Center, Athens, GA, United States; ^3^ Department of Animal and Dairy Science, University of Georgia, Athens, GA, United States

**Keywords:** nutraceutical, gut health, gut microbiota, broilers, microbial homeostasis

## Abstract

Necrotic enteritis (NE) is an enteric disease of poultry that alters the structure of the gut microbial community causing dysbiosis. This 28 day experiment investigated the effects of 125% and 135% arginine diets on the gut microbial diversity and composition of broilers during a subclinical NE challenge. One hundred and twenty one-day-old chicks were randomly allocated to 4 treatments with six replicates each- Uninfected + Basal, NE + Basal, NE + Arg 125%, and NE + Arg 135% diet groups. NE was induced by inoculating 1 × 10^4^ *E. maxima* sporulated oocysts on day 14 and 1 × 10^8^ CFU *C. perfringens* on days 19, 20, and 21 of age. The NE challenge significantly decreased the number of observed amplicon sequence variants (*p* = 0.03), the abundance of the phylum Firmicutes (*p* < 0.01), and the species *Mediterraneibacter cottocaccae* (*p* = 0.01) in the ceca of birds on day 21. The NE challenge significantly increased the Bray-Curtis index (*p* < 0.01), and the abundance of the phylum Bacteroidota (*p* < 0.01), family Odoribacteraceae (*p* < 0.01), genus *Odoribacter* (*p* < 0.01), and species *O. splanchnicus* (*p* = 0.01) on day 21. During NE, the 125% arginine diet restored the abundance of the phylum Bacteroidota (*p* = 0.03), family Odoribacteraceae (*p* = 0.03) and Oscillospiraceae (*p* = 0.03), genus *Odoribacter* (*p* = 0.03), and species *O. splanchnicus* (*p* = 0.03) and *M. cottocaccae* (*p* < 0.01) on day 21. The 135% arginine diet effectively restored the loss in alpha diversity (*p* = 0.01) caused by NE, the abundance of the phylum Firmicutes (*p* = 0.01) and Bacteroidota (*p* < 0.01), family Oscillospiraceae (*p* = 0.03) and Odoribacteraceae (*p* < 0.01), genus *Odoribacter* (*p* < 0.01), and species *O. splanchnicus* (*p* < 0.01) and *M. cottocaccae* (*p* < 0.01) on day 21. On day 28, the treatments had a significant effect on the cecal propionate (*p* = 0.01), butyrate (*p* = 0.04), and total SCFA (*p* = 0.04) concentrations. In conclusion, the 125% and 135% arginine diets restored gut microbial composition during a subclinical NE challenge, but not the cecal SCFA profile. Hence, arginine in combination with other feed additives could be used in restoring gut microbial homeostasis during NE in poultry.

## 1 Introduction

Necrotic enteritis (NE) is caused by the Gram-positive, boxcar-shaped anaerobic bacteria *Clostridium perfringens*. NE causes an estimated annual loss of more than $6 billion US dollars for the global poultry industry (Fathima et al., 2024a). While *C. perfringens* is recognized as the etiological agent of NE, the presence of predisposing factors is essential for the induction of this multifactorial disease complex (Fathima et al., 2022a). The predisposing factors create a conducive environment for the proliferation and colonization of *C. perfringens* by causing damage to the intestinal epithelium, increasing mucus secretion, disrupting the gut microbial homeostasis, altering the gut transit times, and causing immune suppression (Fathima et al., 2022a; Moore, 2016). However, the predisposing factors implicated in triggering NE, such as coccidiosis, mycotoxins, and dietary factors, directly or indirectly alter the poultry gut microbiome, promoting the colonization by pathogenic *C. perfringens* (Lacey et al., 2018).

The gastrointestinal (GI) tracts of humans and animals harbor a myriad of diverse microorganisms, numbering in trillions. Collectively, these microorganisms play a pivotal role in sustaining host health by supplying nutrients and energy, regulating immune responses, and competitively excluding pathogens (Fathima et al., 2022b). A disturbance in the gut microbial homeostasis or dysbiosis has been linked to the occurrence of a variety of enteric diseases (Sun and Jia, 2018). The onset of NE is associated with a shift in the gut microbiota in poultry (Fathima et al., 2022a). Nonetheless, it remains uncertain whether this shift in microbial composition acts as a predisposing factor or if it is predominantly a consequence of the proliferation of *C. perfringens* and the ensuing necrosis. Pathogenic *C. perfringens* interact and compete with other gut microbiota, potentially influencing the initiation and severity of the disease. Therefore, comprehending the reciprocal impact of microbiota composition on *C. perfringens* colonization and toxin production, and vice-versa, is of utmost significance (Antonissen et al., 2016).

In the post-antibiotic era, various alternative strategies for the control of NE in poultry have been proposed and investigated, such as organic acids (Pham et al., 2020), probiotics (Khalique et al., 2020), synbiotics (Shah et al., 2023), plant extracts (Vidanarachchi et al., 2013), and functional amino acids (Hilliar et al., 2020; Zhang et al., 2019). Arginine is a functional amino acid that is essential for poultry species due to the absence of a complete urea cycle. Apart from its role in protein synthesis, arginine is also essential for the synthesis of various low-molecular-weight bioactive molecules such as nitric oxide (NO), ornithine, creatine, polyamines, and agmatine that play a crucial role in various physiologic and immunologic functions (Ren et al., 2014; Fathima et al., 2024b). Hence, arginine modulates gene expression, energy metabolism, protein synthesis, wound healing, growth, and overall health of animals, including poultry (Ren et al., 2014; Dai et al., 2012). Recent studies indicate that arginine may also play a role in regulating the metabolism of intestinal microbiota (Ren et al., 2014). The potential explanation for the regulatory role of arginine supplementation on gut microbiota could be attributed to the alterations in the intestinal microenvironment induced by arginine supplementation. Arginine supplementation has the potential to regulate the utilization and metabolism of amino acids by certain bacterial species (Dai et al., 2012). This, in turn, may have an impact on the composition and activity of other gut microbial species.

While the role of arginine in regulating gut microbiota has been extensively studied in wine production and mice models, there is a paucity of research in poultry models. Given arginine’s capacity to modulate intestinal immune responses and gut microbial composition, it holds the potential for mitigating dysbiosis induced by enteric diseases, such as NE, in poultry. Therefore, this study delves into the impact of two levels of arginine supplementation, 25% and 35% above the concentration in the basal diet, on gut microbial homeostasis in poultry during NE. The effect of 125% and 135% arginine diets on growth performance, intestinal health, and immune response during necrotic enteritis were detailed in our first research article (Fathima et al., 2024a). This article provides additional insights into the cecal microbiome and SCFA concentrations, offering a more comprehensive view of the disease’s impact on the birds. In this study, we employed a subclinical NE model characterized by a significant reduction in growth performance but no significant mortality (Fathima et al., 2024a). Hence, this experiment provides insights into the impact of arginine supplementation on the cecal microbial homeostasis and short-chain fatty acid profile of broilers during a subclinical NE challenge.

## 2 Materials and methods

### 2.1 Ethics statement

All animal protocols used in this study were approved by the Institutional Animal Care and Use Committee of the University of Georgia (AUP: A2021 06-002-A4) and were strictly followed during the duration of the study. The birds were monitored twice daily for symptoms of infection and were humanely euthanized if required. All researchers involved in the care, handling, and sampling of the broilers were trained by the University of Georgia animal care and handling (UGA IACUC 101 course).

### 2.2 Experimental design and diets

A total of 480 one-day-old Cobb-500 male broiler chicks were obtained from the Cobb-Vantress hatchery in Cleveland, GA. The chicks were randomly allocated to the four treatment groups- 1. Uninfected + Basal diet, 2. NE + Basal diet, 3. NE + Arg 125% diet, and 4. NE + Arg 135% diet groups. Each treatment was replicated in six pens with 20 birds per pen and a stocking density of 0.96 birds/sqft. The pen was considered an experimental unit. The birds were reared in floor pens with hardwood shavings as litter material. Temperature and lighting schedules adhered to the Cobb-500 husbandry guidelines. Nipple-type waterers were installed in the pens. Feed and water were available *ad libitum* throughout the experiment.

A two-phase feeding schedule was followed in this study: the starter phase (day 0- day 9) and the grower phase (day 10- day 28) (Table 1). The basal diets were formulated to meet or exceed the nutrient recommendation for Cobb-500 broiler chickens (Cobb-Vantress, 2022). In the NE + Arg 125% and NE + Arg 135% groups, L-Arginine (Dyets, Inc.) was supplemented at 25% and 35%, respectively, in excess of the arginine content in the basal diet. Glycine (Dyets, Inc.) was used to balance the crude protein and nitrogen content of the Uninfected + Basal and NE + Basal diets during both phases.

**TABLE 1:** Composition of starter (days 0–9) and grower (days 10–28) diets.

Ingredients (%)	Basal diet		125% arginine diet		135% arginine diet	
	Starter	Grower	Starter	Grower	Starter	Grower
Corn	54.47	59.66	54.74	59.91	54.76	60.01
Corn-DDGS	2.00	4.00	2.00	4.00	2.00	4.00
Canola meal	2.00	5.00	2.00	5.00	2.00	5.00
Soybean meal	33.00	23.00	33.00	23.00	33.00	23.00
Soybean oil	4.60	4.40	4.60	4.40	4.60	4.40
Dicalcium phosphate	1.85	1.54	1.85	1.54	1.85	1.54
Limestone	0.76	0.72	0.77	0.72	0.77	0.72
Lysine	0.02	0.02	0.02	0.02	0.02	0.02
Methionine	0.21	0.19	0.21	0.19	0.21	0.19
Threonine	0.01	0.03	0.01	0.03	0.01	0.03
Arginine	0	0	0.08	0.31	0.12	0.43
Glycine	0.42	0.78	0.06	0.23	0	0
Sodium bicarbonate	0.20	0.20	0.20	0.20	0.20	0.20
Salt	0.28	0.28	0.28	0.28	0.28	0.28
Vitamin premix^a^	0.10	0.10	0.10	0.10	0.10	0.10
Trace minerals premix^b^	0.08	0.08	0.08	0.08	0.08	0.08
Total	100	100	100	100	100	100
Calculated nutrients and energy						
ME^c^, kcal/kg	2998.55	3065.58	3007.58	3073.07	3011.18	3076.11
Protein, g/kg	228.00	199.96	228.00	199.95	228.00	199.92
Arginine, g/kg	14.35	11.81	17.95	14.77	19.38	15.96
TSAA^d^, g/kg	9.07	8.21	9.08	8.22	9.09	8.23
Calcium, g/kg	9.00	8.00	9.00	8.00	9.00	8.00
Available P, g/kg	4.55	4.00	4.55	4.00	4.55	4.00

^a^ Vitamin mix provided the following per kg of diet: 2.4 mg thiamin-mononitrate, 44 mg nicotinic acid, 4.4 mg riboflavin, 12 mg D-Ca pantothenate, 2.7 mg pyridoxine-HCl, 12 g vitamin B12, 0.11 mg D-biotin, 0.55 mg folic acid, 3.34 mg menadione sodium bisulfate complex, 220 mg choline chloride, 1,100 IU cholecalciferol, 11 IU all-racemic-α-tocopherol acetate, 2,500 IU trans-retinyl acetate, and 150 mg ethoxyquin.

^b^ Trace mineral mix provided the following per kg of diet: 101 mg MnSO_4_.H_2_O, 20 mg FeSO_4_.7H_2_O, 80 mg ZnO, 3 mg CuSO_4_.5H_2_O, 0.3 mg sodium selenite, 0.75 mg ethylenediamine dihydroiodide, and 20 mg MgO.

^c^ Metabolizable energy.

^d^ Total Sulfur Amino acids.

### 2.3 Necrotic enteritis challenge

The birds in the NE + Basal, NE + Arg 125%, and NE + Arg 135% diet groups were orally gavaged with 1 × 10^4^ sporulated *E. maxima* oocysts on day 14 post-hatch and birds in the Uninfected + Basal diet group were orally gavaged with 1 mL phosphate-buffered saline. The *C. perfringens* (CP6) (Kindly donated by Dr. Charles Hofacre, SPRG, Athens, GA) was cultured in thioglycolate broth with resazurin (Sigma-Aldrich^®^, MO). Further, on days 19, 20, and 21, the birds in the NE + Basal, NE + Arg 125%, and NE + Arg 135% diet groups were orally gavaged with 1 mL of the broth culture containing 1 × 10^8^ CFU/mL of CP6. The Uninfected + Basal diet group was gavaged with 1 mL phosphate-buffered saline (Reddyvari, 2020). The *C. perfringens* concentration in the broth culture was confirmed by assessing the optical density and comparing it with a standard growth curve established using the strain before the start of the experiment. The successful induction of the NE model was confirmed by the presence of *E. maxima* oocysts in the feces on day 19, decreased growth performance on days 14–21, and increased intestinal permeability and lesion scores on day 21 in the infected groups, the data of which were previously published (Fathima et al., 2024a).

### 2.4 Sample collection and DNA extraction

On days 21 and 28 post-hatch, one bird per pen was euthanized using CO_2_. Cecal content from one bird per replicate (6 birds per treatment) was collected in a sterile cryovial (5 mL). The samples were frozen immediately in liquid nitrogen. Upon reaching the facility, the samples were transferred and stored in a −80°C freezer until further analysis.

Total DNA was extracted from the cecal content using the QIAamp Fast DNA Stool Mini Kit. The DNA extraction from the cecal content was performed following a hybrid protocol as previously described (Welch et al., 2021). This method utilizes enzymatic and mechanical methods to optimize DNA extraction, maximizing yield. Briefly, 0.35 g of cecal content was transferred into a 2 mL Lysing Matrix E tube (MP Biomedicals LLC, Irvine, CA, United States) containing 1.4 mm ceramic spheres, 0.1 mm silica spheres, and one 4 mm glass bead for facilitating the mechanical disruption of bacterial cells. The QIAGEN vortex adapter for the Vortex-Genie 2 (QIAGEN, Venlo, Netherlands) was used at maximum speed for 10 min to disrupt the cells. QIAamp Fast DNA Stool Mini Kit (QIAGEN, Venlo, Netherlands) was used for enzymatic bacterial DNA extraction. Total DNA was eluted in 50 μL of elution buffer according to the manufacturer’s instructions. Following extraction, the concentration and purity of the total DNA were assessed using a spectrophotometer (Synergy H4 Hybrid Multi-Mode Microplate Reader) along with the Take3 Micro-Volume Plate (BioTek Instruments Inc., Winooski, VT, United States). Specimens containing at least a DNA concentration of 10 ng/μL were preserved at −80°C until the next day. Samples not meeting these criteria were dropped and underwent a new cycle of DNA extraction (Williamson et al., 2022).

### 2.5 DNA sequencing

Following extraction, the DNA samples were shipped to Loop Genomics (San Diego, CA) on dry ice for library preparation and the synthetic long-read sequencing of 16S rRNA variable regions (V1 to V9), as described previously (Callahan et al., 2021). The complete sequencing protocol can be found here. Briefly, the hypervariable region of the 16S rRNA gene was synthetically reconstructed from a series of standard Illumina PE150 reads, yielding approximately 20 gigabytes of data, which were assembled to reconstruct the whole 16S rRNA gene variable region.

### 2.6 Bioinformatics analysis

The sequences were converted to FASTQ files and were imported into QIIME 2. The QIIME DADA2 plugin was used to denoise, dereplicate, and filter out chimeras. The filtered sequences were assigned taxonomies using a pre-trained Naive Bayes classifier, which had been trained on the SILVA 138 SSU database. For further analysis, the sequencing depth was established at 2080 sequences per sample. Subsequently, alpha-diversity metrics were calculated for each sample, including the Shannon Diversity Index, Faith’s Phylogenetic Diversity Index, Pielou’s Evenness Index, and the count of amplicon sequence variants (ASVs) or the number of observed features. Concerning beta diversity, outcomes are presented from the Bray-Curtis dissimilarity index. Furthermore, the relative abundances of individual microbial taxa were assessed (Williamson et al., 2022).

### 2.7 Short-chain fatty acid analyses

The short-chain fatty acid (SCFA) concentrations of the cecal samples were analyzed using the procedure described previously (Lourenco et al., 2020). Briefly, the cecal content was collected on days 21 and 28 in 5 mL sterile cryovials on ice and stored at −80°C until further analysis. For the analysis of SCFAs, the samples were solubilized in water prior to processing (1 part of the sample in 3 parts of water). The solubilized samples were centrifuged at 10,000 × g for 10 min. Subsequently, 1 mL of the resulting supernatant was transferred to a new centrifuge tube, along with 0.2 mL of 25% wt/vol metaphosphoric acid solution, and frozen overnight at −20°C. The following day, upon thawing, the samples were centrifuged again at 10,000 × g for 10 min. The supernatant was then transferred to polypropylene tubes and combined with ethyl acetate in a 2:1 ratio (ethyl acetate to supernatant). Following a 15 s vortex and a 5 min settling period, 600 µL of the supernatant was transferred to screw-thread vials for the analysis of SCFA using a Shimadzu GC-2010 Plus gas chromatograph (Shimadzu Corporation, Kyoto, Japan). The gas chromatograph was equipped with a flame ionization detector and a capillary column (Zebron ZB-FFAP; 30 m × 0.32 mm × 0.25 μm; Phenomenex Inc., Torrance, CA, United States). The sample injection volume was 1.0 μL, and helium served as the carrier gas. The column temperature was initially set at 110°C and gradually increased to 200°C. Injector and detector temperatures were set at 250°C and 350°C, respectively. The peak heights of the samples were compared to those of standards to ascertain the concentrations of SCFAs in the samples.

## 3 Statistical analysis

A one-way analysis of variance (ANOVA) was conducted to examine the impact of arginine supplementation on the SCFA concentration of broilers during NE and were considered significantly different at *p* ≤ 0.05. The means were separated using Tukey’s HSD. The individual microbial taxa and alpha diversity indices were analyzed using the non-parametric Kruskal-Wallis test. Beta diversity differences were evaluated through permutational multivariate analysis of variance (PERMANOVA - Adonis). The results from all statistical tests underwent correction using Bonferroni’s method to address the false discovery rate (FDR) resulting from multiple comparisons. FDR-adjusted *p*-values were deemed statistically significant when *p* ≤ 0.05.

## 4 Results

### 4.1 Effect of arginine supplementation on the alpha-diversity indices

**FIGURE 1:**
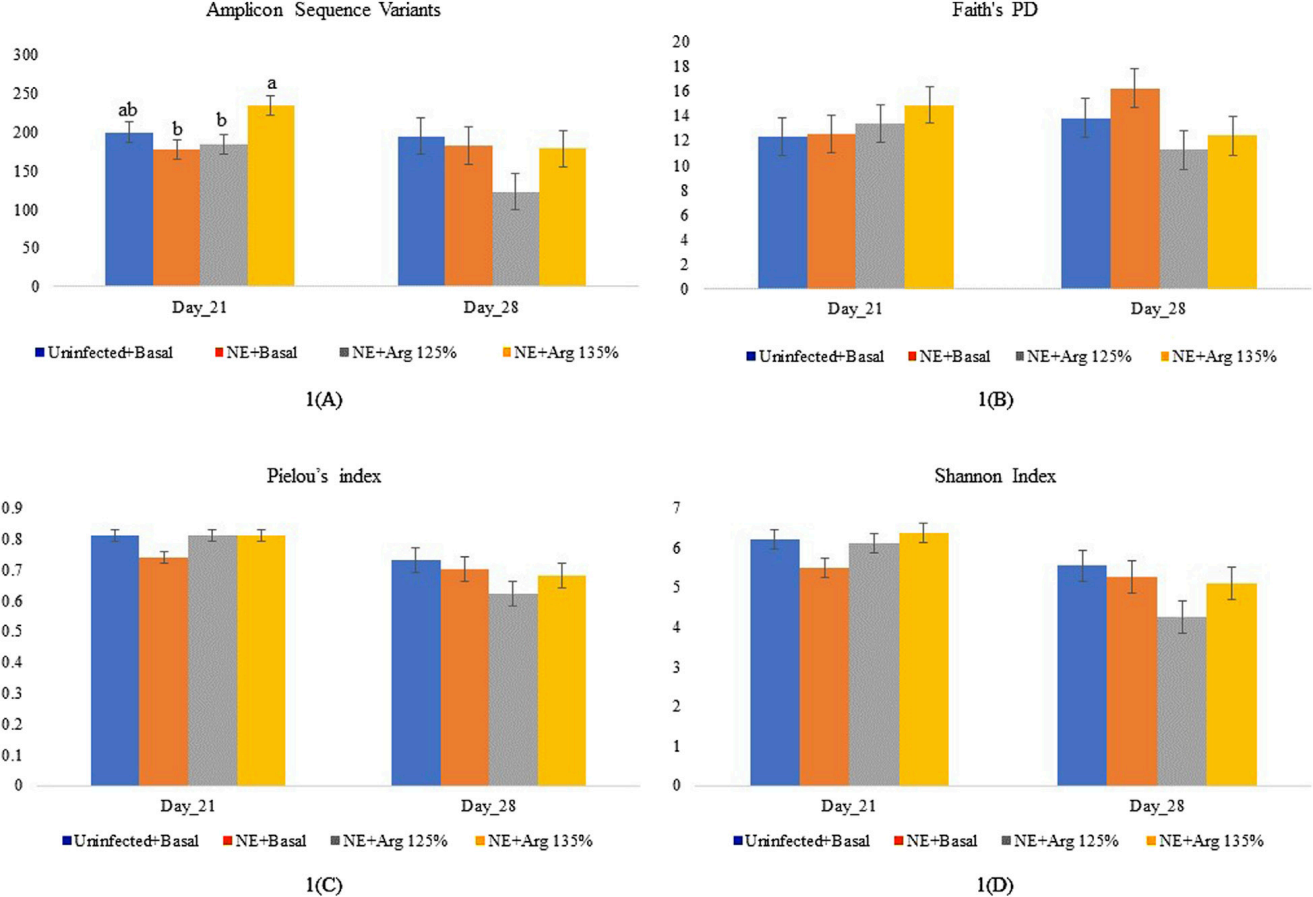
The effect of 125% and 135% arginine diets on the alpha diversity indices on days 21 and 28, **(A)** Amplicon Sequence Variants, **(B)** Faith’s phylogenetic diversity, **(C)** Pielou’s index **(C)**, and Shannon index **(D)**. Birds in the challenge group were orally gavaged with 1 × 10^4^ sporulated oocysts of *E. maxima* on day 14 and 1 × 10^8^ CFU/mL of *C. perfringens* broth culture on days 19, 20, and 21. Cecal samples were collected on days 21 and 28. Following DNA extraction, the variable regions of the 16S rRNA gene were sequenced (V1 to V9). Bars (±SEM) with no common superscript differ significantly (*p* < 0.05).

On day 21, arginine supplementation significantly affected the number of ASVs (*p* = 0.03) (Figure 1A). The NE + Arg 135% group had a significantly higher number of ASVs compared to the NE + Basal diet and NE + 125% arginine diet groups. The NE challenge decreased the number of ASVs in the cecal content of the birds; however, 135% arginine supplementation restored the loss in alpha diversity during NE. However, the NE challenge did not have a significant effect on Faith’s phylogenetic diversity (*p* = 0.72) (Figure 1B), Pielou evenness index (*p* = 0.12) (Figure 1C), and Shannon diversity (*p* = 0.16) (Figure 1D) on day 21 (Table 2). Also, there were no significant differences in the alpha diversity of treatment groups on day 28 (*p* > 0.05).

**TABLE 2:** Effect of 125% and 135% arginine diets on alpha diversity indices during NE.

Alpha diversity indices	Uninfected + basal	NE + basal	NE + 125% Arg	NE + 135% Arg	*p*-value
Day 21					
ASVs	200.00^ab^	177.50^b^	184.50^b^	234.67^a^	0.03
Faith’s PD	12.35	12.53	13.39	14.88	0.62
Pielou’s index	0.81	0.74	0.81	0.81	0.11
Shannon Index	6.20	5.49	6.11	6.38	0.09
Day 28					
ASVs	194.83	182.83	122.83	179.00	0.17
Faith’s PD	13.84	16.27	11.25	12.43	0.17
Pielou’s index	0.73	0.70	0.62	0.68	0.27
Shannon Index	5.54	5.26	4.25	5.10	0.17

Birds in the challenge group were orally gavaged with 1 × 10^4^ sporulated oocysts of *E. maxima* on day 14 and with 1 × 10^8^ CFU/mL of *C. perfringens* broth culture on days 19, 20, and 21. Cecal samples were collected on days 21 and 28. Following DNA extraction, the variable regions of the 16S rRNA gene were sequenced (V1 to V9). On days 21 and 28, the alpha diversity indices were calculated, namely the number of Amplicon Sequence Variants (ASVs), Faith’s Phylogenetic diversity (Faith’s PD), Pielou’s evenness index, and Shannon entropy index. The alpha diversity indices were analyzed using the non-parametric Kruskal–Wallis H test. Different letters in the same row indicate significant differences (*p* ≤ 0.05).

### 4.2 Effect of arginine supplementation on the beta diversity based on Bray-Curtis index

**FIGURE 2:**
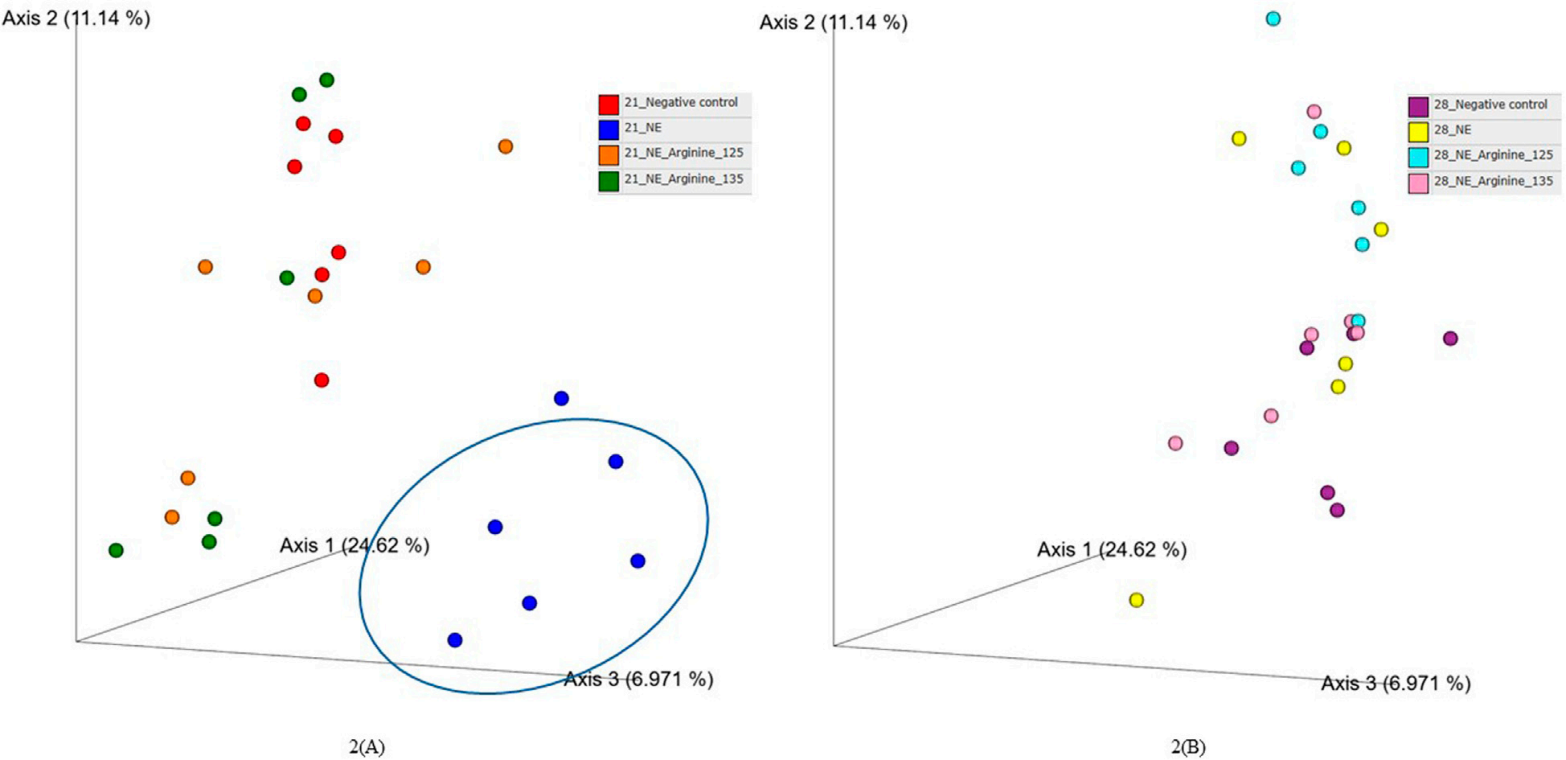
The effect of 125% and 135% arginine diets on the beta diversity (Bray-Curtis dissimilarity index) on day 21 **(A)** and day 28 **(B)**. Birds in the challenge group were orally gavaged with 1 × 10^4^ sporulated oocysts of *E. maxima* on day 14 and 1 × 10^8^ CFU/mL of *C. perfringens* broth culture on days 19, 20, and 21. Cecal samples were collected on days 21 and 28. Following DNA extraction, the variable regions of the 16S rRNA gene were sequenced (V1 to V9). Each dot represents a replicate for each treatment (n = 6).

The beta diversity between the samples was calculated using the Bray-Curtis dissimilarity index, which showed a clear separation between the NE + Basal diet group and other treatment groups (*p* < 0.01) on day 21 (Figure 2A). The differences observed in the alpha diversity were mirrored in the beta diversity, indicating a possible shift in the overall structure of the microbial communities following the NE challenge. From Figure 2A, it can be observed that arginine supplementation significantly reduced the differences in the beta diversity of the microbial communities compared to the NE + Basal group, contributing to a microbial community structure similar to that of the Uninfected + Basal group. However, on day 28, there were no significant differences in the beta diversity for the treatment groups (*p* > 0.05) (Figure 2B). The Adonis test showed a significant effect of the treatments (*p* < 0.01) and days on the beta diversity of the birds (*p* < 0.01) (Table 3).

**TABLE 3:** Effect of 125% and 135% arginine diets on cecal microbiota beta diversity using adonis test.

	DF^a^	SS^b^	MSS^c^	F-value	R[Table-fn Tfn6]	*P*-value
Treatment	3	1.36	0.45	1.88	0.10	0.003
Day	1	1.91	1.91	7.94	0.14	0.001
Treatment: Day	3	1.04	0.35	1.45	0.07	0.052
Residuals	40	9.63	0.24		0.69	
Total	47	13.94			1.00	

Birds in the challenge group were orally gavaged with 1 × 10^4^ sporulated oocysts of *E. maxima* on day 14 and with 1 × 10^8^ CFU/mL of *C. perfringens* broth culture on days 19, 20, and 21. Cecal samples were collected on days 21 and 28. Following DNA extraction, the variable regions of the 16S rRNA gene were sequenced (V1 to V9). On days 21 and 28, the Adonis test was done to determine the effect of the days and treatment on the cecal beta diversity of the birds.

^a^ Degrees of freedom.

^b^ Sum of squares.

^c^ Mean sum of squares.

### 4.3 Effect of arginine supplementation on the cecal microbiota at the phyla level on days 21 and 28

**FIGURE 3:**
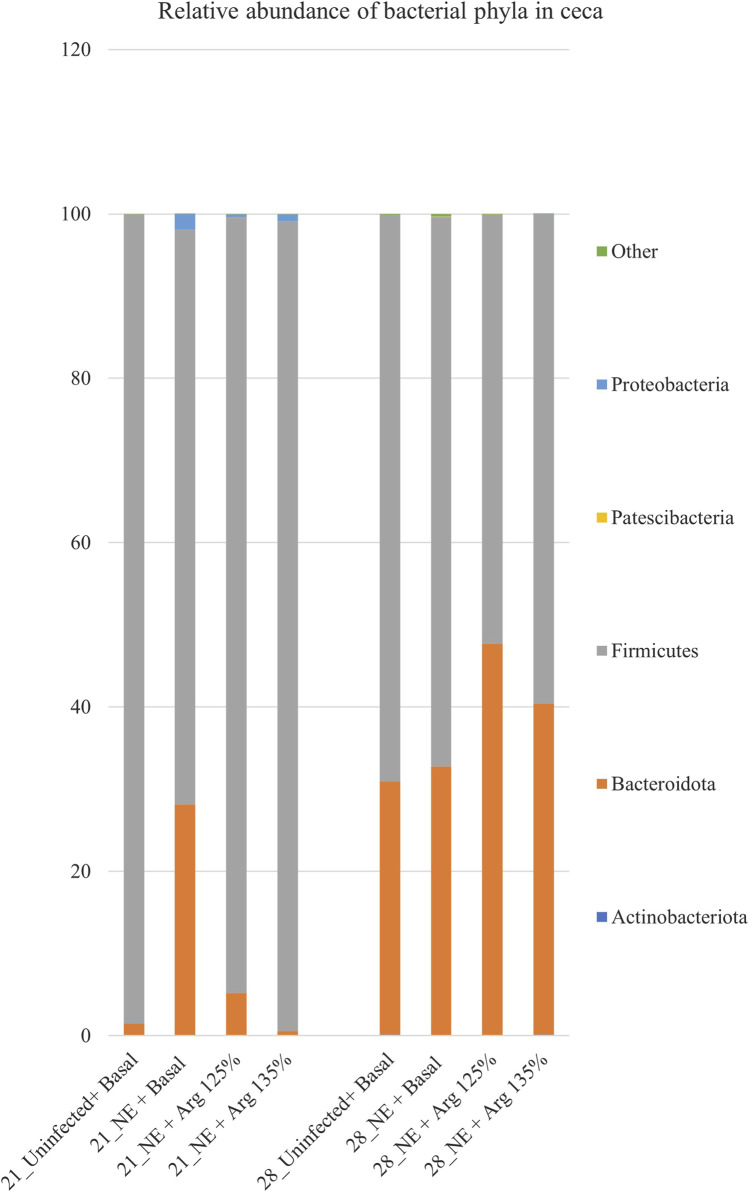
Effect of 125% and 135% arginine diets on the relative abundance of phyla. Birds in the challenge group were orally gavaged with 1 × 10^4^ sporulated oocysts of *E. maxima* on day 14 and 1 × 10^8^ CFU/mL of *C. perfringens* broth culture on days 19, 20, and 21. Cecal samples were collected on days 21 and 28. Following DNA extraction, the variable regions of the 16S rRNA gene were sequenced (V1 to V9). The taxonomic classification was performed using the QIME 2 feature-classifier plugin, which uses the Naïve Bayes classifier trained on the SILVA 138 SSU database. The relative abundance of phyla was analyzed using the Kruskal–Wallis H test and considered significantly different when the FDR-corrected *p* ≤ 0.05.

**FIGURE 4:**
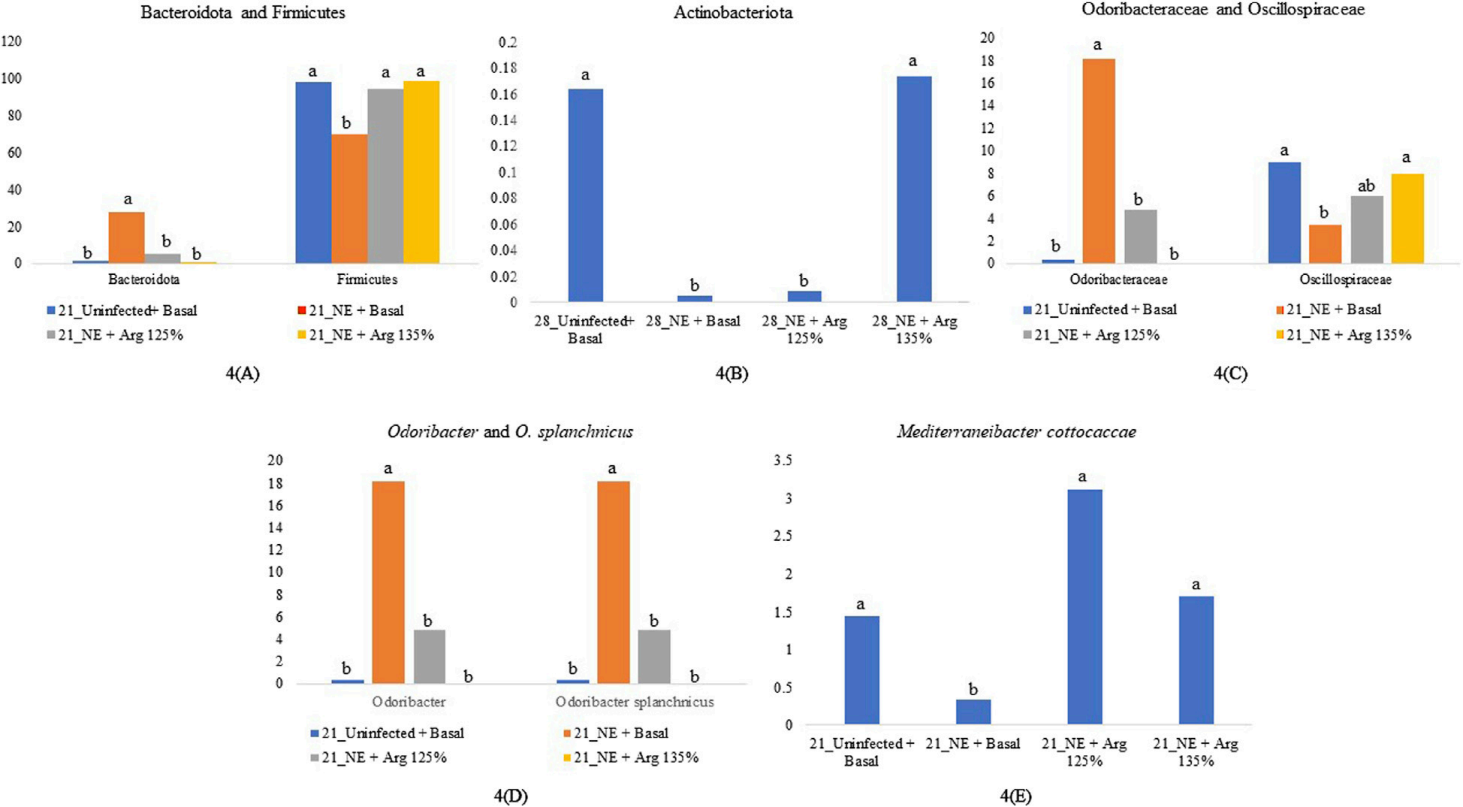
Relative abundance of significantly different taxonomic groups at different levels. Birds in the challenge group were orally gavaged with 1 × 10^4^ sporulated oocysts of *E. maxima* on day 14 and 1 × 10^8^ CFU/mL of *C. perfringens* broth culture on days 19, 20, and 21. Cecal samples were collected on days 21 and 28. Following DNA extraction, the variable regions of the 16S rRNA gene were sequenced (V1 to V9). The taxonomic classification was performed using the QIME 2 feature-classifier plugin, which uses the Naïve Bayes classifier trained on the SILVA 138 SSU database. The relative abundance of taxonomic groups was analyzed using the Kruskal–Wallis H test. Relative abundance of phyla Bacteroidota and Firmicutes on day 21 **(A)**, Relative abundance of phylum Actinobacteriota on day 28 **(B)**, Relative abundance of family Odoribacteraceae and Oscillospiraceae on day 21 **(C)**, Relative abundance of genus *Odoribacter* and species *Odoribacter splanchnicus* on day 21 **(D)**, and Relative abundance of the species *Mediterraneibacter cottocaccae* on day 21 **(E)**. The relative abundance of the individual taxonomic groups was analyzed using the Kruskal–Wallis H test and considered significantly different when the FDR-corrected *p* ≤ 0.05.

After completing all quality control and filtering steps, the sequence counts across our samples ranged from 2,080 to 8,229. A total of 5 phyla were identified in the cecal content. Firmicutes (90.35%) and Bacteroidota (8.77%) were the most dominant phyla on day 21 (Figure 3). On day 21, there was a significant effect of the treatment on the relative abundance of the phylum firmicutes (*p* < 0.01) (Figure 4A). The NE + Basal diet group had a significantly lower abundance of Firmicutes in the cecal content compared to the Uninfected + Basal, NE + Arg 125%, and NE + Arg 135% (*p* < 0.01) treatment groups. There was also a significant effect of the treatment on the abundance of the phylum Bacteroidota (*p* < 0.01) (Figure 4A). The NE + Basal diet group had a significantly higher abundance of the phylum Bacteroidota in their cecal content compared to the Uninfected + Basal, NE + Arg 125%, and NE + Arg 135% diet groups. Similar to that observed for the phylum Firmicutes, there were no significant differences in the relative abundance of Bacteroidetes between the Uninfected + Basal, NE + Arg 125%, and NE + Arg 135% diet groups, implying the potential effect of arginine in restoring the gut microbial composition during the NE challenge. There were no significant treatment effects on the abundance of the phyla Actinobacteriota (*p* = 0.75), Patescibacteria (*p* = 1.00), and Proteobacteria (*p* = 0.24).

On day 28, Firmicutes (61.89%) and Bacteroidota (37.88%) were the dominant phyla in the cecal content (Figure 3). The treatments significantly affected the abundance of phylum Actinobacteriota (*p* = 0.03) (Figure 4B). The abundance of the phylum Actinobacteriota was considerably reduced in the NE + Basal and NE + Arg 125% diet groups, compared to the Uninfected + Basal diet group. However, there were no significant treatment effects on the abundance of Bacteroidota (*p* = 0.55), Firmicutes (*p* = 0.59), Patescibacteria (*p* = 0.55), and Proteobacteria (*p* = 0.44) on day 28.

### 4.4 Effect of arginine supplementation on the cecal microbiota at the family level on days 21 and 28

**FIGURE 5:**
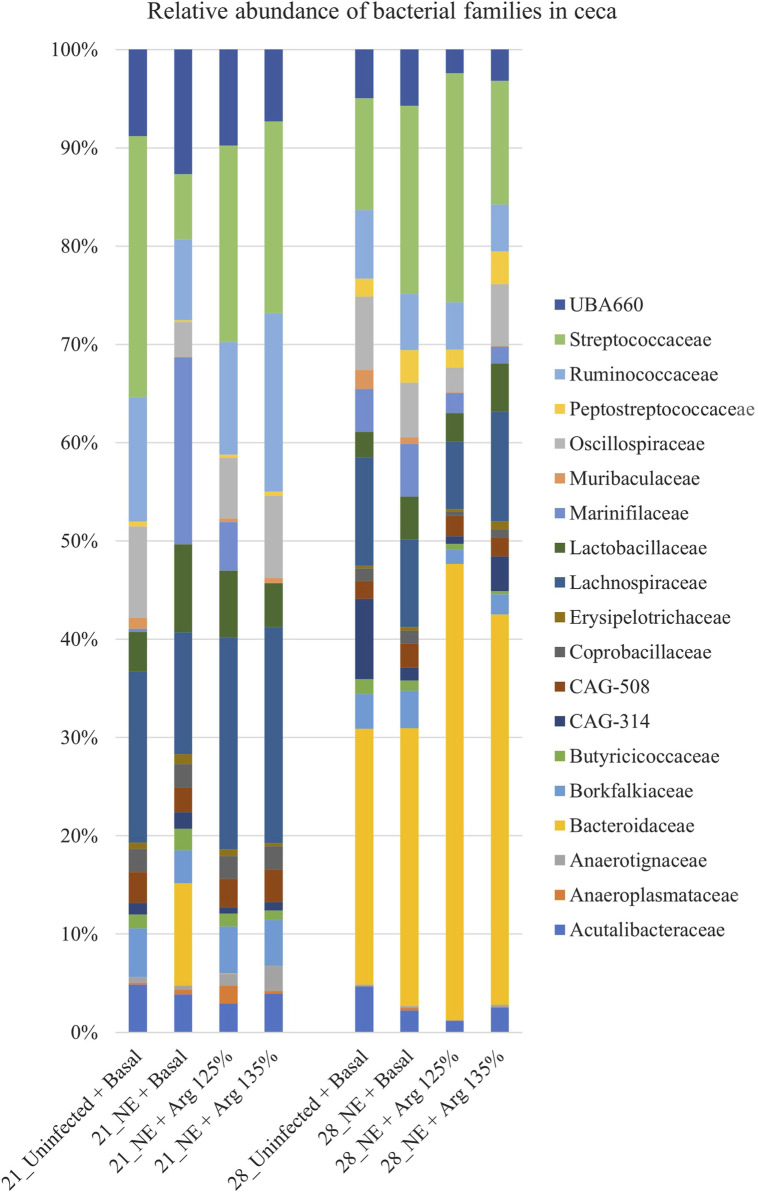
Effect of 125% and 135% arginine diets on the relative abundance of families. Birds in the challenge group were orally gavaged with 1 × 10^4^ sporulated oocysts of *E. maxima* on day 14 and with 1 × 10^8^ CFU/mL of *C. perfringens* broth culture on days 19, 20, and 21. Cecal samples were collected on days 21 and 28. Following DNA extraction, the variable regions of the 16S rRNA gene were sequenced (V1 to V9). The taxonomic classification was performed using the QIME 2 feature-classifier plugin, which uses the Naïve Bayes classifier trained on the SILVA 138 SSU database. The relative abundance of families was analyzed using the Kruskal–Wallis H test and considered significantly different when the FDR-corrected *p* ≤ 0.05.

A total of 51 families were identified in the cecal content of the birds. On day 21, Lachnospiraceae (17.63%), Streptococcaceae (17.49%), and Ruminococcaceae (12.11%) were the dominant families whereas, on day 28, Bacteroidaceae (33.97%), Streptococcaceae (16.04%), and Lachnospiraceae (9.13%) dominated. Bacterial families that constituted more than 0.5% (composing approximately 94% of the bacterial abundance) were analyzed for differences in their relative abundances on day 21 and day 28 (Figure 5).

On day 21, the relative abundance of the family Odoribacteraceae (*p* < 0.01) and Oscillospiraceae (*p* = 0.02) was significantly affected by the treatments (Figure 4C). The abundance of the family Odoribacteraceae was significantly higher in the NE + Basal diet group, compared to the Uninfected + Basal, NE + 125% Arg diet groups, and NE + 135% Arg diet groups (*p* < 0.01). On the other hand, the NE + Basal diet group had a considerably lower abundance of the family Oscillospiraceae compared to the Uninfected + Basal diet group (*p* = 0.01). Interestingly, 135% arginine diet restored the loss in abundance, to a level comparable to that of the Uninfected + Basal diet group. However, no significant effect of the treatments was observed on the abundance of families on day 28.

### 4.5 Effect of arginine supplementation on the cecal microbiota at the genus level on days 21 and 28

**FIGURE 6:**
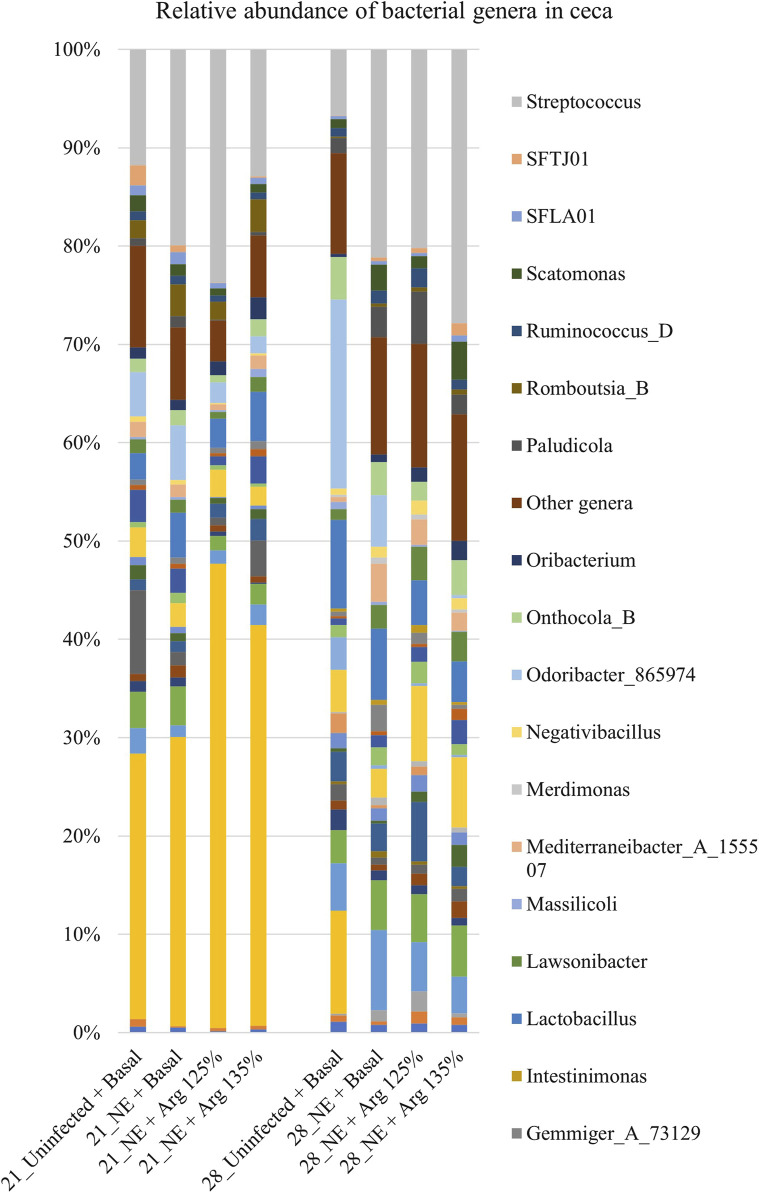
Effect of 125% and 135% arginine diets on the relative abundance of genera. Birds in the challenge group were orally gavaged with 1 × 10^4^ sporulated oocysts of *E. maxima* on day 14 and with 1 × 10^8^ CFU/mL of *C. perfringens* broth culture on days 19, 20, and 21. Cecal samples were collected on days 21 and 28. Following DNA extraction, the variable regions of the 16S rRNA gene were sequenced (V1 to V9). The taxonomic classification was performed using the QIME 2 feature-classifier plugin, which uses the Naïve Bayes classifier trained on the SILVA 138 SSU database. The relative abundance of genera was analyzed using the Kruskal–Wallis H test. The relative abundance of genera was analyzed using the Kruskal–Wallis H test and considered significantly different when the FDR-corrected *p* ≤ 0.05.

A total of 142 genera were identified in the cecal content. Bacterial genera constituting at least 0.5% were analyzed for the relative abundance on days 21 and 28. On day 21, *Streptococcus* and *Odoribacter* were the most abundant genera in the cecal content, whereas *Bacteroides* and *Streptococcus* were the dominant genera on day 28 (Figure 6).

On day 21, there was a significant effect of treatment on the relative abundance of the genus *Odoribacter* (*p* < 0.01) (Figure 4D). The abundance of *Odoribacter* was significantly higher in the NE + Basal diet group, compared to the other treatment groups (*p* < 0.01). The 125% and 135% arginine diets significantly decreased the NE-induced increase in the abundance of the bacterial species *O. splanchnicus*, to a level comparable to that of the Uninfected + Basal diet group. Similarly, a tendency was observed in the effect of the treatments on the relative abundance of the genus *Streptococcus* (*p* = 0.06). The NE + Basal diet group had a substantially lower abundance of *Streptococcus* compared to that of the Uninfected + Basal diet group. 125% arginine supplementation during NE was observed to restore the loss in abundance of *Streptococcus* during NE. However, on day 28, there were no significant treatment effects on the relative abundance of the bacterial species analyzed.

### 4.6 Effect of arginine supplementation on the cecal microbiota at the species level on days 21 and 28

**FIGURE 7:**
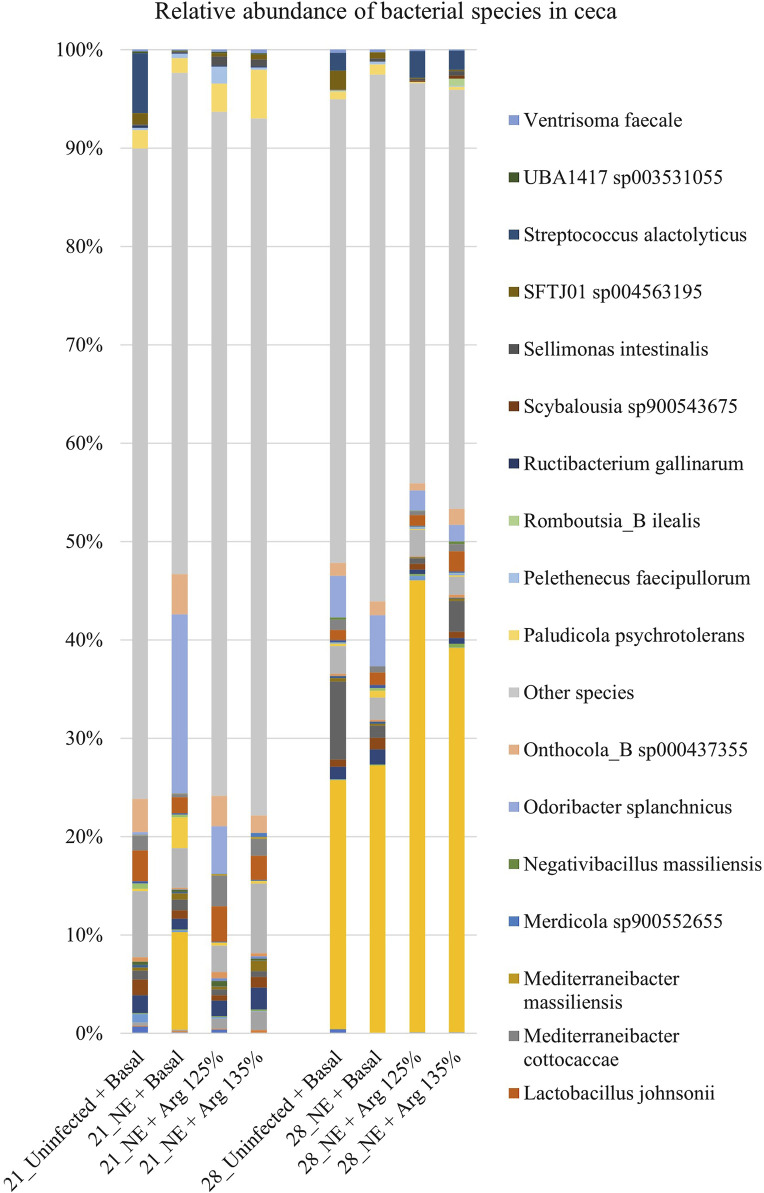
Effect of 125% and 135% arginine diets on the relative abundance of species. Birds in the challenge group were orally gavaged with 1 × 10^4^ sporulated oocysts of *E. maxima* on day 14 and with 1 × 10^8^ CFU/mL of *C. perfringens* broth culture on days 19, 20, and 21. Cecal samples were collected on days 21 and 28. Following DNA extraction, the variable regions of the 16S rRNA gene were sequenced (V1 to V9). The taxonomic classification was performed using the QIME 2 feature-classifier plugin, which uses the Naïve Bayes classifier trained on the SILVA 138 SSU database. The relative abundance of species was analyzed using the Kruskal–Wallis H test. The relative abundance of species was analyzed using the Kruskal–Wallis H test and considered significantly different when the FDR-corrected *p* ≤ 0.05.

A total of 126 species were identified in the cecal content. Bacterial species constituting at least 0.5% were analyzed for the relative abundance on days 21 and 28 and are shown in Figure 7. On day 21, *Odoribacter splanchnicus* and *Faecalibacterium* sp002160895 were the most abundant genera in the cecal content, whereas *Bacteroides fragilis* and *Odoribacter splanchnicus* were dominant on day 28.

On day 21, there was a significant treatment effect on the relative abundance of the species *Odoribacter splanchnicus* (*p* < 0.01) (Figure 4D). The abundance of *Odoribacter splanchnicus* was significantly increased in the NE + Basal diet group compared to the other treatment groups. Similar to that observed for the genus *Odoribacter*, the 125% and 135% arginine diets significantly decreased the NE-induced increase in the abundance of the bacterial species *O. splanchnicus* comparable to that of the Uninfected + Basal diet group. On the other hand, a considerable decrease in the abundance of the species *Mediterraneibacter cottocaccae* was observed in the NE + Basal diet group compared to the Uninfected + Basal diet group (*p* = 0.01) (Figure 4E). As previously observed, 125% and 135% reversed the effect of the NE challenge, decreasing the abundance of the bacterial species during NE to levels comparable to the Uninfected + Basal diet group. However, no significant treatment effects were observed on the relative abundance of the bacterial species on day 28.

### 4.7 Effect of arginine supplementation on the cecal short-chain fatty acid concentrations on days 21 and 28

On day 21, the treatments had a significant effect on the cecal isobutyrate (*p* = 0.04) concentration of the birds. The Uninfected + Basal diet group had a significantly lower cecal isobutyrate concentration compared to the other treatment groups (Table 4). The NE challenge significantly increased the isobutyrate concentration in cecal content. However, the treatments did not have a significant effect on the cecal acetate, propionate, butyrate, isovalerate, valerate, or total SCFA concentrations.

**TABLE 4:** The effect of 125% and 135% arginine diet on the cecal short-chain fatty acid concentrations.

SCFAs^1^ (mM)	Uninfected + basal	NE + basal		NE + Arg 125%	NE + Arg 135%		SEM^2^	*p*-value
Day 21								
Acetate	79.84	89.86		80.89	88.77		6.45	0.59
Propionate	6.16	7.24		6.32	7.77		1.42	0.83
Isobutyrate	0.84^b^	1.37^a^		1.47^a^	1.49^a^		0.17	0.04
Butyrate	11.84	13.53		13.14	14.44		2.28	0.88
Isovalerate	0.78	1.12		1.38	1.20		0.22	0.31
Valerate	1.19	1.74		1.71	1.81		0.20	0.14
Total SCFA	100.64	114.86		104.94	115.60		8.47	0.53
Day 28								
Acetate	82.74	67.58		85.17	66.76		5.75	0.06
Propionate	6.23^ab^	4.28^b^		8.33^a^	6.32^ab^		0.76	0.01
Isobutyrate	0.61	0.74		0.96	0.66		0.09	0.09
Butyrate	13.78^a^	9.58^ab^		13.88^a^	7.88^b^		1.68	0.04
Isovalerate	0.52	0.59		0.85	0.56		0.09	0.10
Valerate	1.32	0.98		1.46	1.01		0.15	0.10
Total SCFA	105.20^ab^	83.77^b^		110.67^a^	83.19^b^		7.73	0.04

Birds in the challenge group were orally gavaged with 1 × 10^4^ sporulated oocysts of *E. maxima* on day 14 and with 1 × 10^8^ CFU/mL of *C. perfringens* broth culture on days 19, 20, and 21. Cecal samples were collected on days 21 and 28. The SCFA, concentrations in the cecal content were assessed using gas chromatography. SCFA concentrations were analyzed using a one-way analysis of variance and Tukey’s HSD was used to determine the difference between groups. Different letters in the same row indicate significant differences (*p* ≤ 0.05). ^1^Short-Chain Fatty Acids, ^2^Standard Error of Mean.

On day 28, the treatments had a significant effect on the cecal propionate (*p* = 0.01), butyrate (*p* = 0.04), and total SCFA (*p* = 0.04) concentrations. The NE + Arg 125% diet group had a significantly higher cecal propionate concentration compared to the NE + Basal diet group. On the other hand, the NE + Arg 135% diet group had a significantly lower cecal butyrate concentration compared to the NE + Arg 125% and Uninfected + Basal diet groups. The acetate concentration tended to be significantly affected by the treatments (*p* = 0.06). The NE + Arg 125% group tended to have a significantly higher concentration of acetate in the cecal content compared to the NE + Basal and NE + Arg 135% diet groups. The total SCFA concentration was significantly higher in the NE + Arg 125% group compared to the NE + Basal and NE + 135% Arg diet groups. The 125% arginine diet was observed to restore the cecal SCFA concentration of acetate, propionate, butyrate, and total SCFAs during NE on day 28 (Table 4).

## 5 Discussion

Necrotic enteritis is a serious gastrointestinal infectious disease that alters the structure of the gut microbial community (Stanley et al., 2014). In poultry, the ceca, characterized by its two blind pouches with a relatively slow passage rate, serves as the optimal habitat for a diverse microbiome that significantly influences host nutrition and health. The cecal microbiome, primarily comprised of bacteria, is extensively researched and stands as the most studied intestinal microbiome in poultry (Pan and Yu, 2014). The cecal microbiome plays a crucial role in preventing pathogen colonization, detoxifying harmful compounds, nitrogen recycling, and microbial fermentation of undigested feed in chickens (Rothhammer et al., 2016; Yan et al., 2017). This beneficial effect is attributed to the production of SCFAs in the cecum through the fermentation of dietary fibers by anaerobic bacteria in the hindgut (Rothhammer et al., 2016). Changes in the composition of the intestinal microbiota have been documented to play significant roles in influencing the metabolism and immune function of the host (Yan et al., 2017). The onset of NE is associated with gut microbial dysbiosis (Fathima et al., 2022a). There are very few investigations into the effect of NE challenge on the cecal microbial homeostasis in broilers (Stanley et al., 2014; Miska et al., 2023; Bortoluzzi et al., 2019). Hence, gaining insight into the alteration in the cecal microbiome and metabolic functions during NE and the effect of arginine supplementation on cecal microbial homeostasis will improve our understanding of the development of NE and devise alternative strategies to prevent NE.

Alpha diversity refers to the variety of species or entities present within a specific sample (Gautam et al., 2024). The evenness, richness, and overall alpha diversity of the cecal content were assessed using the number of ASVs, Faith’s phylogenetic diversity, Pielou’s evenness index, and the Shannon diversity index. A diverse intestinal microbiota composition is beneficial for maintaining the stability of the intestinal microenvironment and protecting against invasion by pathogenic microorganisms (Tang et al., 2022). Similar to that observed in other studies (Bortoluzzi et al., 2019; Yang et al., 2021), a decrease in the number of ASVs in the ceca during NE was observed in this study. The 135% arginine diet restored the number of ASVs of the cecal content on day 21, which reflected that arginine supplementation could prevent microbial dysbiosis during NE. Arginine supplementation is known to restore the microbial community structure, increase the species diversity, and stabilize the community, preventing dysbiosis (Agnello et al., 2017). However, in a study conducted by Zhang et al. (2018), an increase in the alpha diversity of the ileal content was observed during the NE challenge. The observed differences might be due to the differences in the experimental model and conditions and also the intestinal section analyzed (Zhang et al., 2018; Lourenco et al., 2022). There were no significant treatment effects observed on the other alpha diversity indices analyzed, which agrees with the findings of a similar study conducted by Bortoluzzi et al. (2019). The significant difference observed in the number of ASVs, but not in other alpha diversity indices might be because the number of ASVs takes into consideration the rare taxa which might not affect the other diversity indices (Risely et al., 2021).

The beta diversity between the samples was visualized using the principal coordinates analysis (PCoA) plots based on the Bray-Curtis dissimilarity index (Xiao et al., 2021). A clear separation of the NE + Basal diet group was observed from the other treatment groups during the acute phase of NE, which indicates the dissimilarities between microbial communities between the treatments, as observed in previous studies (Miska et al., 2023; Zhang et al., 2020) as well. Interestingly, arginine supplementation reversed the shift in microbial communities caused by the NE challenge, restoring beta diversity to levels comparable to uninfected controls. This finding is consistent with other studies where arginine supplementation in poultry challenged with enteric pathogens like *Salmonella* Typhimurium and *C. perfringens* also restored the gut microbial diversity (Zhang et al., 2018; Zhang et al., 2020), emphasizing arginine’s role in promoting microbial resilience under stress.

Similar to other study reports, the abundance of Firmicutes was significantly decreased during NE (Zhang et al., 2018; Xu et al., 2018). Arginine supplementation was previously reported to reverse microbial dysbiosis during NE in poultry, in line with the study results (Zhang et al., 2018). In contrast, the NE challenge significantly increased the cecal abundance of Bacteroidota during the acute phase, similar to those observed in a similar study on cecal microbial abundance during NE (Bortoluzzi et al., 2019). Bacteroidota are important contributors to intestinal health, that can inhibit the proliferation of *C. perfringens* (Wrigley, 2004). A decrease in the abundance of Bacteroidota is associated with the incidence of NE in poultry, caused by declined intestinal health (Xu et al., 2023). Arginine supplementation during NE, however, reinstated the microbial balance similar to those observed in the uninfected group. The NE challenge also decreased the relative abundance of Actinobacteriota during the recovery phase. Similar findings were observed in a study conducted by Yang et al. (2021), where the abundance of the phylum was found to be correlated with the severity of NE in poultry. The phylum Actinobacteriota is positively associated with the maintenance of intestinal homeostasis in poultry (Ouyang et al., 2023). During NE, the intestinal dysbiosis caused by *Eimeria* and *C. perfringens* might have contributed to the reduced abundance of Actinobacteriota in poultry (Dittoe et al., 2023).

At the family level, the abundance of Oscillospiraceae was considerably reduced by the NE challenge. A similar trend was reported by other studies examining the effect of NE challenge on gut microbial composition (Yang et al., 2021). Oscillospiraceae are positively correlated with gut microbial diversity (Falalyeyeva et al., 2023; Ameer et al., 2023). Arginine supplementation may have restored the abundance of *Oscillospiraceae* through the synthesis of nitric oxide (NO), a molecule that modulates vascular tone, immune responses, and cellular signaling pathways (Wu et al., 2009). NO production can enhance blood flow to the gut, improving nutrient absorption and creating a more favorable environment for the growth of butyrate-producing bacteria (Moncada and Higgs, 2006).

At the family, genus, and species level, the NE challenge significantly increased the relative abundance of Odoribacteraceae, *Odoribacter*, and *O. splanchnicus*, respectively. The functional role of the family *Odoribacteraceae* in poultry remains poorly characterized in the existing literature. The genus *Odoribacter* in poultry may be associated with gut microbial instability as the abundance of *Odoribacter* was observed to decrease significantly as age increases and in healthy birds (Xi et al., 2019). The microbial instability caused by NE was resolved by arginine supplementation, bringing down the abundance of *Odoribacter*, comparable to that of the uninfected group. A significant increase in the species *O. splanchnicus* was observed corresponding to the increased abundance of genus *Odoribacter*. *O. splanchnicus*, previously known as *Bacteroides splanchnicus* was observed to possess immunoregulatory activity in human subjects (Shah et al., 2017). The increased abundance of *O. splanchnicus* in poultry during NE might be due to the gastrointestinal inflammation and dysbiosis (Shang et al., 2016) caused by NE. Interestingly, arginine supplementation resolved the increase in the abundance of the species during NE, decreasing the abundance of the species in the cecum (Hiippala et al., 2020). Arginine supplementation can alter the availability of nutrients in the gut, particularly those required for the growth of specific bacterial taxa (Wu et al., 2009). *Odoribacter* is known to ferment complex polysaccharides to produce SCFAs, including propionate and butyrate (Shang et al., 2016). Arginine can influence the metabolism of other gut bacteria, which may outcompete *Odoribacter* for available substrates when arginine is supplemented. This competitive exclusion could have led to a reduction in the abundance of *Odoribacter* (Dai et al., 2020).

Conversely, a decrease in the abundance of the genus *Streptococcus* and species *Mediterraneibacter cottocaccae* was observed during NE. A similar decline in the abundance of *Streptococcus and Mediterraneibacter* was also observed in a study conducted by Yang et al. (2021), which was closely correlated with the severity of NE in poultry (Yang et al., 2021). *Mediterraneibacter* plays a significant role in promoting gut health through the production of SCFAs in the gut (Lin et al., 2023). During NE, commensal species such as *Mediterraneibacter* will be replaced by the proliferation of pathogenic species such as *C. perfringens*, leading to a decreased abundance of those beneficial gut bacteria. Remarkably, the 125% arginine diet restored the abundance of *Streptococcus* during the NE challenge. This could be because arginine supplementation favors the proliferation of *Streptococcus* species, which further aids in neutralizing gut pH imbalances and restoring microbial balance (Agnello et al., 2017).

Short-chain fatty acids are gut microbial fermentation end-products (Abdelli et al., 2020). Among the SCFAs measured, acetic acid, butyric acid, and propionic acid were the major SCFAs detected. Similar to that observed in this study, an increased concentration of isobutyrate and propionate during NE has also been reported in other studies (Sharma et al., 2018; Gharib-Naseri et al., 2019). The increased concentration of these SCFAs during NE can be due to the rapid growth and fermentation of *C. perfringens*, producing isobutyrate and propionate (Sharma et al., 2018). The increased isobutyrate concentration could also be due to the *Eimeria* challenge (Liu et al., 2023). However, the cecal concentrations of acetate and butyrate were observed to decrease during the NE challenge. These observations are in agreement with the results of previous studies conducted by Gharib-Naseri et al. (2019) and Kumar et al. (2022). Most of the acetate and butyrate producers belong to the phylum Firmicutes (Emami et al., 2020; Liu et al., 2021). The NE challenge significantly reduced the abundance of Firmicutes in the cecal content, which substantiates the reduced cecal acetate and butyrate concentrations during NE. Also, the NE challenge significantly reduced the abundance of the major butyrate producer Oscillospiraceae (Hao et al., 2021), which could also have possibly contributed to the decreased cecal butyrate concentration. It is noteworthy that while significant microbial shifts were observed during the acute phase of the infection, the SCFA concentrations in the infected birds did not show substantial changes compared to the control group. This may be attributed to the SCFAs being actively utilized in inflammatory responses during this phase, thereby limiting their accumulation despite the altered microbial composition (Liu et al., 2021; Kles and Chang, 2006). Nevertheless, there is very little data on the effect of NE on the cecal SCFA concentration during the recovery phase. During the recovery phase, though there weren’t significant changes in the cecal microbial composition between the treatment groups, the SCFA composition differed. In general, the 125% arginine diet tended to increase the concentration of propionate, butyrate, and total SCFA concentrations attributable to possible diet-challenge interaction. This could be due to the alterations in the metabolic pathways of the cecal microbiota in birds fed the 125% arginine diet.

The findings of this study complement the previously published work on the effects of 125% and 135% arginine supplementation on growth performance, intestinal health, and immune responses in broilers during NE (Fathima et al., 2024a). The earlier study reported that while arginine did not reverse the NE-induced loss in production performance, it significantly enhanced immune responses, including increased bile IgA concentration and T-cell differentiation. The current study’s results demonstrate restoration of microbial diversity and partial recovery of the SCFA profile, suggesting arginine’s benefits extend to gut microbiota regulation, which may contribute to the improved immune responses observed (Salim et al., 2016).

Moreover, the increase in the expression of immune response markers such as inducible nitric oxide synthase and interferon-γ in the 125% arginine group aligns with the observed microbial shifts, as these immune responses are often linked to gut microbial activity and composition (Fathima et al., 2024b; Wu et al., 2000). Thus, the combined effects of arginine on both immune modulation and gut microbiota highlight its potential as a multifaceted approach to managing subclinical NE in broilers.

## 6 Conclusion

Increased diversity of intestinal microbiota might have a protective effect from enteric diseases. Arginine supplementation effectively restored the loss in gut microbial diversity caused by the necrotic enteritis challenge and maintained gut microbial homeostasis in poultry. In this study, 135% arginine supplementation was observed to have a more beneficial effect in relation to restoring gut microbial homeostasis compared to the 125% arginine-supplemented group during NE. However, further research is required to determine the mechanisms by which arginine exerts its effects on the gut microbiota during enteric diseases in poultry.

## Data Availability

The microbial sequence data generated and analyzed during the current study have been deposited in the NCBI Sequence Read Archive (SRA) and are publicly available under the accession number PRJNA1149910. These data can be accessed at the following URL: https://www.ncbi.nlm.nih.gov/sra/PRJNA1149910. Additional details regarding the data processing and analysis can be provided upon reasonable request.
